# The Potential Use of ChatGPT as a Sensory Evaluator of Chocolate Brownies: A Brief Case Study

**DOI:** 10.3390/foods14030464

**Published:** 2025-02-01

**Authors:** Damir D. Torrico

**Affiliations:** Department of Food Science and Human Nutrition, University of Illinois Urbana-Champaign, Urbana, IL 61801, USA; damir@illinois.edu; Tel.: +1-217-300-0396

**Keywords:** ChatGPT, sensory characteristics, chocolate brownies, sentiment analysis, product development

## Abstract

ChatGPT, a recently developed natural large language processing tool, has been widely explored in various fields of science and research. This study aimed to evaluate the potential use of ChatGPT as a sensory evaluator of hypothetical formulations of chocolate brownies. ChatGPT was prompted to act as an experienced taster to provide a detailed description of sensory characteristics for fifteen chocolate brownie formulations grouped into three categories (standard, common ingredients replacements, and uncommon ingredients replacements). Sentiment analysis, emotions/descriptors classification, and correspondence analysis were conducted to analyze ChatGPT responses. Results showed that the terms “trust”, “anticipation”, and “joy” were the most frequently expressed sentiments in the ChatGPT responses. The valence of all ChatGPT responses was mostly positive. The overall quality scores of all chocolate brownie formulations given by ChatGPT were extremely high, in the range of 8.5–9.5 (out of 10). ChatGPT tended to have higher positive emotions to formulations (some including worm meals and fish oil) that might have the opposite reactions with real consumers. Further research should focus on validating ChatGPT sensory descriptors with the outcomes of a human sensory panel. Additionally, future studies can explore the potential use of ChatGPT in evaluating the sensory characteristics of other food products for optimizing the product development process.

## 1. Introduction

ChatGPT [1] is a state-of-the-art natural large language processing tool that uses artificial intelligence models to generate human-like conversations [2]. This innovative technology has been shown to have several useful applications, including the generation of blogs, summaries, programming codes, and other text-based outputs in multiple fields of science and research [3]. For instance, one of the most practical uses of ChatGPT is in the tourism industry, where it has been explored as a tool to enhance customer service and increase productivity and efficiency in back-of-house operations [4]. In education, ChatGPT is highly useful for summarizing complex content; however, concerns have been raised regarding its impact on honesty and truthfulness in written assessments by students [5]. Nonetheless, the availability of this technology to generate text built on knowledge-based content can be beneficial in terms of screening information for different purposes.

Food product development relies on sensory evaluation by experts or trained panels [6]. This process can be lengthy and expensive. Therefore, researchers are looking for alternatives to screen the sensory characteristics/notes of a wide range of products without running extensive and costly panel sessions. In previous studies, artificial intelligence and machine learning modeling have been used to predict the sensory characteristics of products based on physicochemical measurements [7]. A recent study investigated the cross-modal correspondences (sensory interactions) captured by ChatGPT-3.5 and -4o, focusing on taste associations with shapes and colors across different languages. This study showed that significant language- and version-specific variations generated by ChatGPT aligned with human patterns, but these lacked the intrinsic variability observed in human studies [8]. Schlechter [9] discussed that artificial intelligence (AI) and large language models such as ChatGPT have been progressively integrated into the brewing industry with several practical applications, including development and consumer customization. A comprehensive review that explored the use of AI in sensory and consumer studies found that the majority of recent studies focused on predicting modeling using machine learning algorithms and computer vision [10]. Using these disruptive technologies can profoundly change the process of developing new products in the future.

Despite the application of ChatGPT in various fields, to date, no research has explored the use of this technology as a potential evaluator of food products for sensory screening purposes. Thus, this brief study aimed to evaluate ChatGPT as a generator of sensory descriptions and characteristics for several hypothetical formulations of chocolate brownies. The experiment aimed to cover a broad range of sensory outcomes by creating formulations with changes in percentages and substitutions of ingredients.

## 2. Materials and Methods

### 2.1. Stimuli

For the ChatGPT [1] (version 3.5) experiment, fifteen hypothetical formulations of chocolate brownies were used as stimuli (Table 1). Brownies were selected as food models based on previous experiments in our research group that consisted in altering common formulation with uncommon ingredients [11]. A base formulation, named formulation 1 (F1), was developed according to the recommendation of a standard chocolate brownie recipe given by ChatGPT. This formulation (F1) consisted of chocolate (30%), flour (15%), sugar (20%), butter (25%), and eggs (10%). Then, fourteen additional variation recipes were created from this initial base formulation, changing selected percentages and ingredients. All formulations were classified into three main categories: (1) standard formulations (having changes in the percentages of the base ingredients), (2) common replacements formulations (changing some of the ingredients with common replacements), and (3) uncommon replacements formulations (changing some of the ingredients with uncommon replacements). The variations in formulations aimed to cover a broad range of sensory outcomes based on the differences in the ingredients and their percentages.

### 2.2. ChatGPT Prompts

After creating the hypothetical recipes/formulations, ChatGPT was prompted with two instructions for each of the fifteen formulations. Prompt 1: “*Act as an experienced taster; the following text will give you the ingredients to make a chocolate brownie; use these ingredients to create a brownie. Based on the ingredients, pretend that you are tasting the product and describe the sensory characteristics. Be specific, mentioning the main sensory notes and intensities you can detect. Do not mention the ingredients or the steps, just the sensory notes. The brownie has the following ingredients and percentages: Variable 1*”. In this prompt, Variable 1 was the formulation of one of the fifteen chocolate brownies (Table 1). An additional prompt/instruction was used to ask ChatGPT about the overall sensory score of the chocolate brownie. Prompt 2: “*Act as an experienced taster, give a quality score (out of 10, 0 being a very poor quality and 10 being a very high quality) of the brownie based on the following description of its taste; just provide the numerical value: Variable 2*”. In this prompt, Variable 2 was the ChatGPT response from Prompt 1. Both prompts were used for all fifteen formulations (Table 1).

ChatGPT responses were generated using a Google Sheets extension (Alphabet, Mountain View, CA, USA) that automated the evaluation process. Prompts describing each brownie formulation were input into spreadsheet cells, and ChatGPT’s responses were produced in adjacent cells. This standardized approach ensured consistency in evaluations, avoiding potential biases from using multiple chat interfaces. Formulations were grouped into three categories (standard, common ingredient replacements, and uncommon ingredient replacements), and responses were pooled within each category for analysis.

### 2.3. Statistical Analysis

For analyzing the output/responses given by ChatGPT, the procedure described by Chen et al. [12] was used. The text from the ChatGPT response (from the individual formulations) was analyzed using Natural Language Processing (NLP) techniques, which included text segmentation, sentence tokenization, lemmatization, and stemming. Additionally, keyword frequency counts, sentiment analysis, and emotion classification were performed using the R package ‘syuzhet’ (Version 1.3.1093, Free Software Foundation, Boston, MA, USA). Word count and other data visualization techniques were applied using packages such as ‘ggplot2’ and ‘wordcloud’ in R (Version 1.3.1093, Free Software Foundation, Boston, MA, USA). To investigate the relationship between emotions (from the sentiment analysis) or descriptive terms and the different chocolate brownie formulations, correspondence analysis and multiple factor analysis were conducted using the XLSTAT software (Version 2018.1.1.62926, Addinsoft Inc., New York, NY, USA) in Excel, with an alpha = 0.05.

## 3. Results and Discussion

Table 2 shows the sentiment analysis conducted on the ChatGPT responses regarding the different chocolate brownie formulations (Table 1). In general, the terms “trust”, “anticipation”, and “joy” were the most frequently expressed sentiments found in the ChatGPT responses. On the other hand, “disgust”, “fear”, and “trust” were the least frequently expressed sentiments in these responses. The valance of the ChatGPT responses was predominantly positive (positive counts = 12–23 vs. negative counts = 4–8). In addition, the ChatGPT overall quality score for all brownies was in the range of 8.5–9.5. These scores are extremely high, considering that ChatGPT was instructed to use a 10-point scale to its full extent. However, there was a marginal difference (although not significant; *p* ≥ 0.05) in the scores from the uncommon replacement formulations compared to the standard and common replacement formulations (8.5–9.5 vs. 9.0–9.5). Positive valence towards foods, including those with uncommon elements that may typically be perceived negatively by regular consumers, suggests that ChatGPT exhibits hedonic asymmetry in food evaluation analogous to that observed in humans. This phenomenon indicates that the model tends to respond with significantly positive biases, particularly when addressing unconventional food items, reflecting a positively skewed assessment strategy in its evaluations. Such findings align with existing literature exploring hedonic factors’ influence on consumer food preferences and evaluation processes (e.g., Varela et al. [13]; Leong et al. [14]; Schifferstein and Desmet [15]).

**Figure 1:**
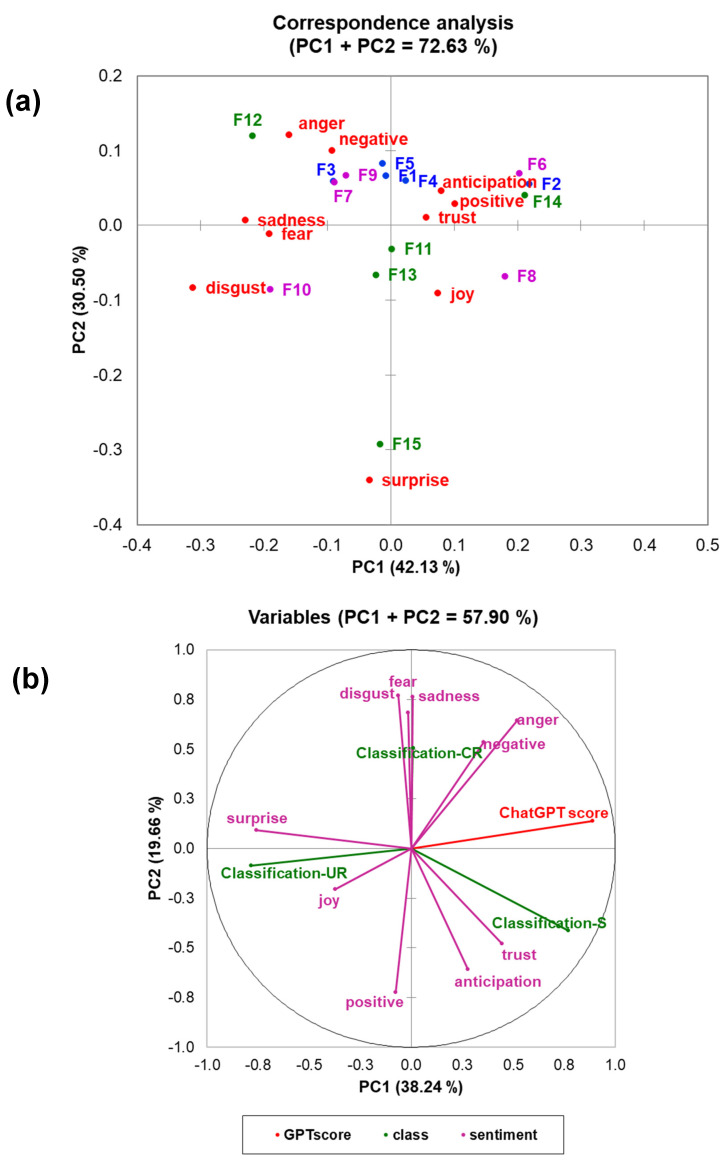
(**a**) Correspondence analysis of the sentiment terms obtained from the ChatGPT responses that were associated with the different chocolate formulations, and (**b**) multiple factor analysis of the sentiment terms and the ChatGPT quality score associated with the different chocolate formulations *. * Formulation descriptions (F1–F15) are provided in Table 1, S = Standard, CR = Common replacements, and UR = Uncommon replacements.

There were also notable findings when analyzing the sentiments of the responses given by ChatGPT. Figure 1a shows the correspondence analysis (CA) of the sentiment terms obtained from the ChatGPT responses associated with the different chocolate brownie formulations. The CA PC1 (42.13%) and PC2 (30.50%) accounted for 72.63% of the total variability in the biplot. A group of standard formulations [F1 (the base standard formulation), F4, and F5] were associated with “anticipation”, “positive”, and “trust” sentiments. On the other hand, the common and uncommon replacement formulations (F6-F15) were mainly separated across the PC1 of the biplot. Interestingly, the F10 (a common replacement formulation with the largest change compared to the base standard formulation F1) was associated with “disgust”. Additionally, F15 (the most uncommon formulation of the set) was associated with “surprised”.

Figure 1b shows the multiple factor analysis (MFA) of the sentiment terms and the ChatGPT quality score (out of 10) associated with the different chocolate formulations. The MFA PC1 (38.24%) and PC2 (19.66%) accounted for 57.90% of the total variability in the biplot. The standard formulations (S) were associated with sentiments “trust” and “anticipation”, common replacements formulations (CR) were associated with the sentiments “disgust”, “fear”, and “sadness”, and the uncommon replacements formulations (UR) were associated with the sentiment “surprised”. The MFA also shows that the ChatGPT overall quality score was negatively associated with the term “surprised” and the uncommon replacement formulations. In the context of sentiment analysis, ChatGPT showed behavioral similarities to typical consumer responses toward various formulations of brownies. The inclusion of unconventional ingredients, such as fish oil and worm meal, can elicit surprise and other emotions in participants, stemming from the novel nature of these products [16]. This suggests that ChatGPT may incorporate the concept of product familiarity in its evaluations when assessing foods with differing ingredient profiles.

The sentiment analysis used as a standardized tool to interpret ChatGPT’s responses to hypothetical chocolate brownie formulations, categorizing the emotional valence (positive, neutral, or negative) of the generated descriptive terms, has limitations. While this approach provided valuable insights into the emotional and sensory connotations expressed by ChatGPT, it is inherently limited by the predefined lexicon and algorithms of the sentiment analysis model. These limitations highlight the need for further validation using alternative modeling systems and comparative evaluations with human sensory panels. Future research should explore methodologies to assess the contextual background of ChatGPT’s responses, advancing our understanding of its potential as a tool for sensory evaluation and product development.

**Figure 2:**
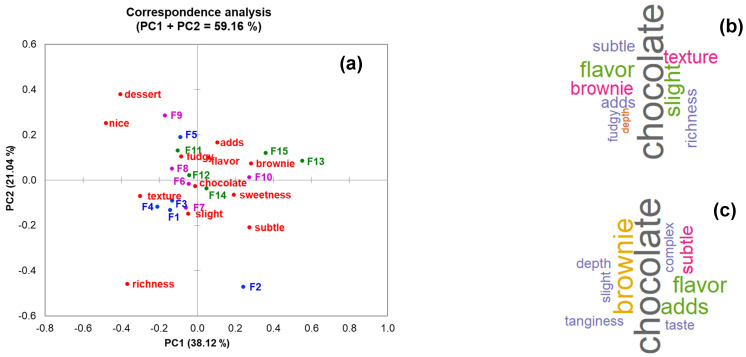
(**a**) Correspondence analysis of the description terms given by ChatGPT, which were associated with the different chocolate formulations, (**b**) word cloud analysis of the description terms given by ChatGPT regarding F1 *, and (**c**) word cloud analysis of the description terms given by ChatGPT regarding F15 *. * Formulation descriptions (F1–F15) are provided in Table 1.

Figure 2a illustrates the correspondence analysis (CA) of the description terms associated with different chocolate brownie formulations provided by ChatGPT (Table S1). The CA PC1 (38.12%) and PC2 (21.04%) collectively accounted for 59.12% of the total variability in the biplot. Specifically, a group of standard formulations [F1 (the base standard formulation), F3, and F4] were characterized by the terms “texture” and “slight”. On the other hand, a group of common replacement formulations (F6–F8) were linked to descriptors such as “chocolate”, “fudgy”, and “flavor”.

Interestingly, F15, the most unconventional formulation of the set, was associated with the term “brownie”. Additionally, Figure 2b,c shows the word cloud analysis of F1 (base standard formulation) and F15 (the most uncommon formulation of the set), respectively. The term “chocolate” was the most frequently used descriptor for both formulations, and there were no major differences in the frequencies of other descriptors. However, the terms “flavor” and “slight” were the second most dominant descriptors for F1, while “brownie” was the second most frequent descriptor for F15.

Future research could explore the integration of established sensory lexicons with ChatGPT to evaluate formulations for specific sensory attributes and liking/preference. By leveraging predefined sensory terminology, ChatGPT’s capability to rank or rate formulations based on intensity or preference could be systematically tested. Such an approach has the potential to streamline product development by providing rapid, AI-driven insights that complement traditional sensory evaluation methods. This direction offers a promising avenue for further exploring the utility of artificial intelligence in sensory science and product optimization.

ChatGPT generated mostly positive sensory outcomes for all the tested hypothetical chocolate brownie formulations. Even for the formulations with uncommon substitutions, such as worm meal, fish oil, and citric acid, ChatGPT had mainly positive comments about these hypothetical foods. The training data used to develop artificial intelligence models should represent a wide range of opinions, perspectives, and experiences in society [17]; otherwise, there is a risk of information bias in the responses. Food, in general, tends to be biased to favorable terms and emotions in the existing text content that can be found in books, websites, articles, and social media [18]. This can be one of the reasons why ChatGPT tended to have positive emotions and sentiments toward foods that might have the opposite reactions from real consumers. The present study shows a preliminary analysis of ChatGPT capabilities for food product formulations. More research is needed to understand how this tool can be used in future sensory evaluation and consumer research applications. Specifically, additional research can involve developing the brownie formulations described in this study and evaluating them with a trained sensory panel. The sensory descriptors and emotional responses generated by the panel should be compared with ChatGPT’s outputs to assess agreement in valence, sensory characteristics, and acceptability. This comparative approach will provide critical insights into ChatGPT’s reliability and applicability. These considerations highlight the need for continued research to bridge AI-generated outputs with human sensory data for broader adoption in food science.

In terms of resources used for running sensory evaluation tests, consumer panels are generally costly, with expenses varying depending on the number of products tested and the nature or complexity of those products [19]. While descriptive analysis is comparatively less expensive, it still requires considerable time to obtain reliable and accurate results [20,21]. Both methods are widely used in the food industry as complementary tools, providing valuable insights into the sensory attributes that drive consumer preferences [22]. Conversely, large language models such as ChatGPT can be used in a single session, potentially reducing the costs and time associated with sensory evaluations. However, the output generated by ChatGPT must be validated to ensure its reliability and to develop predictive models capable of generating responses that align closely with human perceptions. Despite requiring an initial investment of time to train these models, ChatGPT has the potential to serve as an efficient pre-screening tool for product evaluation, significantly accelerating the sensory analysis process.

## 4. Conclusions

This study explored the potential of using ChatGPT as a generator of sensory descriptions and characteristics for several hypothetical formulations of chocolate brownies. Results showed that ChatGPT was able to provide descriptions of sensory characteristics and quality scores for the different brownie formulations, with sentiment analysis revealing positive emotions expressed in the responses. This finding shows that ChatGPT was positively biased when evaluating the chocolate brownie formulations. Further research should focus on validating ChatGPT sensory descriptors with the outcomes of a human sensory panel. Overall, this study suggests that applying innovative technologies such as ChatGPT can profoundly change the process of developing new food products, reducing the time and costs involved in sensory evaluation.

## Figures and Tables

**Table 1:** Formulations (including ingredients and their respective percentage of the total formulation weight) of chocolate brownies tested in the ChatGPT prompt.

Formulation Classification	Formulation ID	Ingredients	% of the Total Recipe
Standard	F1	Chocolate	30%
		Flour	15%
		Sugar	20%
		Butter	25%
		Eggs	10%
	F2	Chocolate	15%
		Flour	30%
		Sugar	20%
		Butter	25%
		Eggs	10%
	F3	Chocolate	30%
		Flour	25%
		Sugar	10%
		Butter	25%
		Eggs	10%
	F4	Chocolate	30%
		Flour	38%
		Sugar	10%
		Butter	13%
		Eggs	10%
	F5	Chocolate	30%
		Flour	30%
		Sugar	10%
		Butter	25%
		Eggs	5%
Common replacements	F6	Chocolate	30%
		Corn flour	15%
		Sugar	20%
		Butter	25%
		Eggs	10%
	F7	Chocolate	30%
		Flour	15%
		Stevia	20%
		Butter	25%
		Eggs	10%
	F8	Chocolate	30%
		Flour	15%
		Sugar	20%
		Olive oil	25%
		Eggs	10%
	F9	Chocolate	30%
		Flour	15%
		Sugar	20%
		Butter	25%
		Lecithin	10%
	F10	Chocolate	30%
		Corn flour	15%
		Stevia	20%
		Olive oil	25%
		Lecithin	10%
Uncommon replacements	F11	Chocolate	30%
		Corn starch	15%
		Sugar	20%
		Butter	25%
		Eggs	10%
	F12	Chocolate	30%
		Flour	15%
		Citric acid	20%
		Butter	25%
		Eggs	10%
	F13	Chocolate	30%
		Flour	15%
		Sugar	20%
		Fish oil	25%
		Eggs	10%
	F14	Chocolate	30%
		Flour	15%
		Sugar	20%
		Butter	25%
		Worm meal	10%
	F15	Chocolate	30%
		Corn starch	15%
		Citric acid	20%
		Fish oil	25%
		Worm meal	10%

**Table 2:** Sentiment analysis (including basic emotions and valance) and the overall quality score of chocolate brownies tested in the ChatGPT prompt.

Formulation Classification	Formulation *	Anger	Anticipation	Disgust	Fear	Joy	Sadness	Surprise	Trust	Negative	Positive	ChatGPT Score *
Standard	F1	5	8	3	3	7	2	3	10	8	18	9.5
	F2	2	5	1	1	4	1	2	7	4	15	9.5
	F3	5	6	3	3	5	2	3	9	7	15	9.5
	F4	5	9	3	3	8	2	3	12	8	18	9.5
	F5	6	8	3	3	7	2	3	12	8	18	9.0
Common replacements	F6	2	4	1	1	6	1	1	6	4	13	9.0
	F7	5	6	3	3	6	2	3	9	8	15	9.0
	F8	4	9	2	2	9	1	5	11	6	21	9.0
	F9	5	7	3	3	6	2	3	8	8	16	9.0
	F10	5	6	4	3	7	3	5	8	8	13	9.5
Uncommon replacements	F11	5	7	3	3	9	2	4	9	7	18	9.0
	F12	4	4	3	2	3	2	2	4	7	12	9.5
	F13	5	9	4	4	8	3	6	10	8	23	8.5
	F14	2	6	0	1	4	1	3	5	6	14	9.0
	F15	3	6	4	3	9	2	8	10	6	17	8.5

* Formulations descriptions are provided in Table 1. ChatGPT score was a quality score provided by ChatGPT on the tested chocolate brownies using a 10-point scale.

## Data Availability

The data supporting this study’s findings are available from the corresponding author upon reasonable request.

## Supplementary Materials

The following supporting information can be downloaded at: https://www.mdpi.com/article/10.3390/foods14030464/s1, Table S1: Frequency of sensory description terms generated by ChatGPT.
