# Supplementary material for: The Potential Use of ChatGPT as a Sensory Evaluator of Chocolate Brownies: A Brief Case Study
Source: Foods. 2025 Feb 1;14(3):464. doi: 10.3390/foods14030464 (PMC11816367; doi:10.3390/foods14030464)
Supplement: Supplementary file 1 [file foods-14-00464-s001.zip › foods-3410640-supplementary.pdf]

Table S1. Frequency of sensory description terms generated by ChatGPT

Formulation classification	Formulation*	chocolate	flavor	slight	brownie	texture	adds	richness	subtle	fudgy	sweetness	dessert	nice
Standard	F1	11	6	6	5	5	4	4	4	3	2	2	2
	F2	5	2	2	3	1	1	2	3	0	2	0	0
	F3	8	4	4	4	3	3	3	2	2	2	2	1
	F4	9	3	3	3	5	1	2	2	2	3	2	1
	F5	8	5	3	5	3	3	1	1	2	2	2	2
Common replacements	F6	6	3	1	3	3	2	1	1	1	1	0	1
	F7	9	4	4	4	4	3	2	2	2	3	0	2
	F8	7	4	3	6	5	3	2	2	2	2	2	2
	F9	8	5	2	4	3	4	1	2	2	2	3	3
	F10	4	2	3	6	3	2	0	2	1	3	1	0
Uncommon replacements	F11	6	3	2	3	2	2	1	1	1	2	2	1
	F12	6	2	2	4	2	2	1	1	1	1	0	2
	F13	7	5	2	8	0	3	0	3	2	3	0	0
	F14	5	2	1	3	2	2	1	1	1	2	0	1
	F15	7	4	2	5	1	4	0	3	1	2	1	0

* Formulations descriptions are provided in Table 1.
